# To the Editors of the Medical and Physical Journal

**Published:** 1804-07-01

**Authors:** R. O. Millett

**Affiliations:** Hayle, Cornwall


### *To the Editors of the Medical and Physical Journal.*

GENTLEMEN,

I Beg leave to communicate the following observations on the case of fatal diarrhœa which appeared in your Medical and Physical Journal of last month, signed Academicus.

It must be allowed, that medical men who are in the habits of visiting patients, have a peculiar advantage over those who reside at a distance, in discovering the cause and manner of removing every disease; and had not the symptoms in the present case been so well defined, and such as have frequently occurred to me, I should not have ventured to submit my remarks on the subject.

It is very well known that during the period of teething, children are subject to such symptoms as affected the child in question. Various other causes may also occasion similar complaints, and the application of cold may produce them, or aggravate the indisposition.

In the present instance, pudding was generally allowed, and a little good Port wine occasionally, (doubtless from the best intention); but as the former generally contains the white of eggs, and as the latter, however good in quality, seems to be by no means adapted for assisting the digestion of an infant, I conclude both had a tendency to disturb the digestive process, especially as his diet was plentiful; and possibly the child might have been too often permitted to make use of improper things, which parents, from affection, suppose he might be indulged in with impunity.

I think it very difficult for any man to say what was the real cause of the child's illness; I generally attribute such complaints to the presence of indigested sordes and accumulation of slime, which occasion symptoms similar to worms, such as rubbing the nose, &c.; an interruption or regurgitation of bile are also frequently produced by the same cause, and similar symptoms follow; but dissection would have been the most probable means of satisfying the anxious enquirer. Having had a great number of children under my own eye, affected with very similar complaints, of different ages, from three months to three years and older, perhaps Academicus may not think it amiss, if I mention the mode of cure which I have generally proved to be successful, though he did not require it.

 An

An emetic was generally given first, then calomel with resin of jalap, scammony or rhubarb; these were repeated as often as the case required, sometimes daily, though the evacuations might be much in quantity, especially if other symptoms did not abate, if the stools continued greenish, or corrupt. When parents, from timidity, would not consent to have an emetic given in season, I have frequently found it indispensable, though alvine excretions were copious and the stomach not accustomed to urge; small doses of prepared chalk and tinct. opii camph. have sometimes been needful to restrain excessive action of the bowels and to alleviate pain, and sometimes mild glysters have removed hard balls of fæculent matter, by which means the cough and oppression proceeding from the state of the stomach and bowels have abated, the natural secretions have been restored, the yellowness sometimes attendant, removed, and the usual vigour and rosy appearance regained, though their desponding parents supposed them to be almost exhausted by a consumption of a very different nature. Such disorders sometimes occur to children while under inoculation; and if fatal, the deaths of the patients are improperly attributed to inoculation.

I take this opportunity to mention, that I inoculated with the same cow-pox virus three children in April last, about the same time, in different parishes; the first had a very distinct chicken-pox during the second week from the time of inoculation, it being prevalent at the time, and the two others, during the second week from inoculation, had the measles with their usual symptoms, the contagion at the time being very diffusive; the inoculated part on the arms of the children had also the usual appearance and degree of inflammation; neither of the children were dangerously ill.

I am, &c.

*Hayle, Cornwall,*
*June* 11, 1804.

R. O. MILLETT, jun.

